# The Relationship of Micro and Macro Worries on the Acceptability of Corruption: The Role of Basic Human Values

**DOI:** 10.11621/pir.2026.0202

**Published:** 2026-06-01

**Authors:** Ekaterina V. Maklasova, Alexander N. Tatarko, Klaus Boehnke

**Affiliations:** a HSE University, Moscow, Russia; b Bremen International Graduate School of Social Sciences of the Constructor University, Germany

**Keywords:** corruption, worry, values, corruption risk, crime

## Abstract

**Background:**

Corruption severely threatens societal stability and well-being, with various factors contributing to its prevalence. This study, relying on theory and empirical research on the relationship between suffering and fraud, offers a new explanation, that worries may be an essential factor in the acceptability of corruption. People’s worries are the consequences of suffering they experience throughout their lives.

**Objective:**

To investigate the role of basic human values in the relationship between micro/macro worries and the acceptance of corruption.

**Design:**

In the cross-sectional online research, 1,380 Russians participated. The socio-psychological survey includes the revised Portrait Values Questionnaire, a modified version of Kubiak’s method for assessing the acceptability of corruption, and a modified version of Boehnke’s method for assessing worries.

**Results:**

The results demonstrate that micro worries lead to acceptance of corruption, while macro worries induce rejection of corruption. The interaction of values and worries affects the acceptability of corruption and shows that increasing micro worries, against the backdrop of the values Conservation and Self-Transcendence, can lead to high risk of everyday corruption. We observe the opposite situation when macro worries, against the backdrop of the clearly expressed value Openness to Change, lead to decreased corruption risks.

**Conclusion:**

This research contributes theoretically by explaining how negative social phenomena like corruption can be understood through the relationship between suffering and dishonest behavior.

## Introduction

Corruption is one of the most severe threats to the stability and well-being of contemporary societies. Corruption is defined as abusing official power or authority for personal gain (Lambsdorff, 2006).

This study investigates how people’s worries influence their acceptance of corruption. It is important to note that the research results indicate that different-level factors can determine corruption. Macro factors of corruption include climatic conditions (Tatarko et al., 2022), the political and economic environment (Sumah, 2017), professional ethics, legislative activity (Sumah, 2017), the ethnocultural characteristics of society (Lee & Guven, 2013; Sumah, 2017), the size of the public sector in the economy and the availability of economic freedoms (Graeff & Mehlkop, 2003), the level of income inequality (Jong-Sung & Khagram, 2005), the quality of the legislature and competitive economy (Svensson, 2005). The factors of corruption at the individual level include a person’s values (Bosco, 2016; Kravtsova et al., 2017; Tatarko et al., 2020) and moral characteristics (Kreikebaum, 2008; Tanner et al., 2022). Also, the acceptability of corruption is influenced by the level of education, income, gender, age, and marital status (Gupta et al., 2002; Heyneman, 2004; Torgler & Valev, 2006; 2010). The age of an individual has a consistent influence on the acceptability of corruption. The older an individual gets, the less often he/she justifies corruption (Torgler & Valev, 2006).

Relying on theory and empirical research on the relationship between suffering and fraud, we offer a new explanation, that people’s worries may be an essential factor in the acceptability of corruption. People’s worries are the consequences of suffering they experience throughout their lives.

### Relationships Among Suffering, Dishonest Behavior, and Corruption

A person’s choice to participate in corrupt activities may be related to various factors, such as moral principles, the significance of the intended benefit, and societal pressure. At the same time, the participants in corrupt activities are not always dishonest people who approve of corruption. An individual’s choice in favor of corruption in a given situation does not always mean that the person is generally tolerant or has a positive attitude toward corruption. His/her behavior may be conditioned by circumstances that reduce the moral costs of dishonest behavior and allow him/her to act unethically. In addition, individuals may continue to perceive themselves as honest. This is because sometimes the pressure of situational factors outweighs moral principles and values, as shown in classical psychological studies (Milgram, 1963; Zimbardo, 1969). Suffering can act as such a situational factor.

The relationship between unethical behavior and suffering is deeply rooted in many cultural and religious practices. For example, in Christianity and Islam, the idea of religious fasting means that through suffering caused by deprivation and restrictions, people achieve spiritual purification from their sins; that is, suffering redeems behavior that is unethical from a religious point of view.

Also, psychological research supports the relationship between suffering and unethical behavior. Studies show that guilt over immoral actions can lead individuals to inflict physical pain on themselves to alleviate guilt (Bastian et al., 2011; Bastian et al., 2014; Inbar et al., 2013; Nelissen & Zeelenberg, 2009). Conversely, experiencing pain can sometimes justify subsequent dishonest behavior.

Research has shown that a state of social or mental pain can increase a person’s tendency to behave dishonestly (Poon et al., 2013). The mechanism of this relationship can be summarized as follows: Most people believe that people get what they deserve in life (Furnham, 2003). When people experience suffering, they tend to regard it as undeserved. To compensate for such injustice, a person can resort to dishonest or unethical behavior, justifying that behavior by suffering. Thus, people are more likely to behave dishonestly if they perceive a situation as unfair to themselves. In this case, unethical behavior is seen as a way of restoring justice and is therefore perceived as justified in the current situation (Kouchaki & Desai, 2015). For example, patients suffering from chronic pain have a heightened sense of injustice. In this regard, they are trying to restore justice by expanding the boundaries of what is permissible for themselves (McParland & Eccleston, 2013; Sullivan et al., 2012).

In other words, suffering can contribute to a higher tolerance towards dishonest behavior, including justifying corruption. Limited research has been devoted to the relationship between suffering and corruption at the individual level. It has been shown that there is a link between patient suffering and corruption in the healthcare sector. It was found that in a corrupt healthcare system, the chances of receiving high-quality treatment and alleviating the suffering of sick people are often determined by whether the patient will be involved in corrupt activities. This phenomenon is observed in various countries, particularly India (Chattopadhyay, 2013) and Russia (Tereshonok, 2016). Physical suffering or illness makes a sick person or their loved ones more tolerant of corruption to reduce this suffering.

Suffering is quite closely related to anxieties. It is rather difficult to say which is primary, anxiety or suffering, if we are talking about a broad social context. However, anxiety about one’s future or the health of loved ones can definitely lead to suffering, which, in turn, as discussed earlier, can be associated with dishonest behavior, in particular justifying corruption. Let us take a closer look at the research on the relationship between anxiety and suffering.

### Relationship Between Anxiety, Worry, and Suffering

Available research suggests that anxiety can cause a person to worry. For example, a comparative study of people with different levels of anxiety showed that among those who have a higher level of anxiety, worrying is also more pronounced (Eysenck, 1984). Next, worrying was considered one of the critical signs of a person’s anxiety state (Dugas et al., 1997). This is because people with a pronounced state of anxiety predominantly experience negative emotional states (Palmer et al., 2021). An excessive focus on negative emotions increases worrying intensity (Dugas et al., 1997).

It is worth noting that the duration of maintaining a worry state depends on life experiences. The more disturbing information is stored in a person’s memory, the more he/she experiences worrying in the present (Eysenck, 1984).

At the same time, the negative experience can worsen both psychological and physical health. For example, health worries based on past life experiences can worsen a person’s subjective perception of their physical health and increase the number of complaints about their physical condition (Filipkowski et al., 2010). Remembering pain experienced in the past can increase the intensity of physical pain in the current moment and increase worrying about pain in the future (Lefebvre et al., 2017). At the same time, worrying is a condition focused solely on the problem and not on overcoming it. This is primarily due to past negative experiences, which lead to a loss of faith in a person’s ability to cope with a problem (Dugas et al., 1995; Siddique et al., 2006).

Thus, past negative experiences shift the focus of a person’s attention to the problem and form the habit of experiencing the predominantly negative psychological state called anxiety, which can result in worry.

Suffering is a strong emotional reaction caused by something threatening a person’s self or integrity about their physical, emotional, or mental state (Hickman et al., 2004). The history of Russia (wars, revolutions, drastic changes in the structure of society) is associated with much suffering, and consequently, worries remain in people’s collective memory. That is why we are conducting this research in Russia. We suggest that these concerns may predict the acceptability of corruption at the household level. Consequently, anxiety, as a cause or consequence of suffering, may be associated with dishonest behavior, particularly the approval of corruption.

### Micro and Macro Worries. Research Hypotheses

Boehnke and colleagues (1998) distinguished micro and macro worries depending on who or what is the object of an individual’s concern. Micro worries are directly related to the individual or his/her immediate social environment (family, friends, acquaintances). Macro worries are worries about society, the country, and the world. In this case, the object of concern is entities external to the individual. Micro worries are negatively associated with life satisfaction and overall mental health. This dependence is a complex multidirectional relationship.

On the one hand, micro worries can cause a poor psychological state (Boehnke et al., 1998). The more a person worries about personal problems or those related to their loved ones, the worse they adapt to the circumstances and the higher the adverse effects on their psychological well-being (Molina & Borkovec, 1994; Tallis et al., 1994). On the other hand, in some cases, micro worries are a consequence of poor mental health. For example, depressed people are more acutely concerned about various problems (Boehnke et al., 1998).

Thus, micro worries are often associated with the presence of psychological problems. So, having micro worries, a person experiences suffering. Suffering, as previously shown, increases the likelihood of immoral behavior. Therefore, it is acceptable to assume that micro worries are positively associated with the acceptability of corruption.

*H1. Micro worries positively relate to a person’s acceptance of corruption.*

As for macro worries, studies show that concerns about problems external to the individual and his/her close social environment are positively associated with the subjective well-being of the individual and indicate good psychological wellbeing (Griffin & Prior, 1990). This is because people in a bad psychological state, as a rule, are immersed in solving their personal problems. Therefore, they do not have the energy to pay attention to macro-level problems. On the contrary, those people who do not experience psychological problems have more psychological resources to address the outside world and are more concerned about its problems (Hamilton et al., 1989).

Thus, micro worries are associated with solving one’s problems and suffering. In order to avoid suffering, a person may engage in dishonest behavior. In the case of macro worries, a different mechanism is at work. A person is more likely to worry about the situation in the country and the world; accordingly, anything that negatively affects the country, including corruption, is unacceptable. Conversely, corruption can be quite acceptable for a person indifferent to the country’s situation and not caring about its future. Guided by these considerations, a hypothesis about the negative relationship between macro worries and the acceptability of corruption can be articulated.

*H2. Macro worries negatively relate to the acceptability of corruption.*

### Basic Values and Attitude Towards Corruption

Values include the basic principles and beliefs regarding what is desirable, essential, and shared by most people. They guide means of action and ideals of behavior across situations. According to Schwartz’s theory of values (Schwartz, 1992), they are defined as motivational and cross-situational goals that serve as guiding principles in people’s lives. In its original version, the theory described ten basic human values: power, achievement, hedonism, stimulation, self-direction, universalism, benevolence, tradition, conformity, and security.

Schwartz’s (1992) original theory postulates a circular order of values, primarily based on the opposition or the compatibility between certain sets of values. The values can be represented in a two-dimensional structure of four higher-order values in two bipolar value sets. The first set consists of the Openness to Change values, encompassing self-direction and stimulation values, versus the Conservation values that include security, conformity, and tradition values, reflecting a conflict between values towards change and a voluntary self-restriction and preservation of traditional practices and defenses. The second set is Self-Transcendence values, which include benevolence and universalism values, versus self-enhancement values, which include power and achievement values. This higher-order set of values reveals a conflict between accepting other people as equals and concern for their welfare on the one hand, and focusing on individual success and dominance on the other (Schwartz et al., 2012). Schwartz’s value model has been extensively tested in cross-cultural studies.

Values serve as reference points in the social space. They affect society’s attitude towards corruption and are essential in explaining corrupt behavior (Davis & Ruhe, 2003; Husted, 1999; Park, 2003). Establishing values associated with intolerance to corruption is essential, as this is the most important preventive measure against corruption in society (Mansilla, 2003; Welzel et al., 2003).

The basic values of representatives of different cultures are related to the attitudes toward corruption in these cultures, as shown by the example of Russia, Germany, France, and Latvia (Tatarko & Mironova, 2016). Higher-order values are also related to attitudes towards corruption. This was shown by the example of Greece and Russia (Tatarko et al., 2020). Accordingly, since, from a psychological point of view, basic human values are an essential factor related to an individual’s attitude to corruption.

*RQ. Can basic personality values influence the relationship between worries and attitudes to corruption?*

This study examines the relationship between micro/macro worries and attitudes towards corruption, while considering higher-order basic human values.

## Methods

### Participants

In this study, 1,380 subjects took part (34.7% men) between the ages of 18 and 72 (*M* = 39.1, *SD* = 11.1). About 64% of respondents have tertiary education. The level of income of 91% of respondents is higher than the established minimal level.

### Procedure

The cluster strategy with equal allocation was used to select the sample. The study was conducted on the online platform Anketolog.ru. The respondents received a letter inviting them to participate in the study through the platform’s internal services. The invitation contained information about the study’s initiator, its purpose, a request to participate in the study, the confidentiality of information received, the estimated time spent completing the survey (~30 minutes), and a reward for completing the survey for 40 rubles.

The socio-psychological survey includes a block of situations describing various types of everyday corruption to assess the acceptability of corrupt behavior, a block of statements to assess the severity of worries regarding different areas of life, a block of statements to assess the importance of higher-order values, and a block with questions about the socio-demographic characteristics of respondents. The questionnaire was filled out individually, without time restrictions or control by the researcher.

### Materials

*The Acceptability of Corruption (α = .895, M = 2.01, SD = .82)*. A modified version of Kubiak’s method for assessing the acceptability of corruption was used (Kubiak, 2001; Tatarko & Mironova, 2015; Tatarko et al., 2022). The method contains eight situations in which different types of corruption are described. The respondents need to assess the acceptability of these situations on a 5-point Likert scale from “not acceptable” to “acceptable.” For example, “a civil servant hires a family member or friend to work for a government agency, rather than another candidate with higher qualifications” (Tatarko & Mironova, 2015).

*Micro and Macro Worries (α = .893, M = 3.08, SD = .77 / α = .924, M = 3.09, SD = .80).* To measure worries, we used a method based on the classification of worries proposed by Boehnke and his colleagues and adapted using a Russian sample (Boehnke et al., 1998). It allows us to assess two groups of worries. The first group is associated with the individual and their immediate environment. The second group is related to society. The method includes 33 statements describing objects of experience and is organized according to a 4-point Likert scale from 1 (“I do not worry at all”) to 4 (“I am very worried”). For example, “I worry about my country being involved in hostilities” (Boehnke et al., 1998).

*The Higher-Order Values.* To assess the higher-order values, the revised Portrait Values Questionnaire was used (Schwartz et al., 2012), which allows us to assess four higher-order values: Openness to Change (Cronbach’s *α* = .725, *M =* 4.6, *SD =* .58), Self-Transcendence (*α* = .812, *M =* 4.64, *SD =* .62), Self-Enhancement (*α* = .765, *M =* 3.89, *SD =* .73), and Conservation (*α* = .791, *M =* 4.33, *SD =* .64). The questionnaire includes 57 statements that describe certain people for whom respondents have to indicate how similar these are to them on a 6-point Likert scale from “not at all like me” to “very much like me.”

### Data processing

The statistical package Jamovi 2.3.21.0 was used to process the data. Hierarchical regression analysis was applied using the Linear Regression module in Jamovi to assess the relationship between worries and the acceptability of corruption, considering age, education, and income. Moderation analysis was performed using the Linear Regression module in Jamovi by including interaction terms to determine the role of higher-order values in the relationship between macro and micro worries of Russians and the acceptability of corruption.

## Results

The Pearson correlation coefficient analysis revealed statistically significant positive correlations between the acceptability of corruption and micro worries, alongside negative correlations with macro worries, higher-order values, age, and income. Additionally, statistically significant positive intercorrelations were observed among worries and higher-order values. The relationships between socio-demographic variables and key study constructs demonstrated multifaceted patterns: age was found to be significantly and negatively correlated with micro worries, the values of Openness to Change and Self-Enhancement, while showing negative associations with the values of Self-Transcendence and Conservation. Education was negatively related to micro worries and positively to macro worries and age. Income displayed a statistically significant negative correlation with worries, alongside positive associations with the values of Openness to Change and Self-Enhancement (see *Table 1*).

**Table 1:** Pearson Correlation Coefficients Among Acceptability of Corruption, Micro Worries, Macro Worries, Higher-Order Values, and Socio-Demographic Variables

Variables	1	2	3	4	5	6	7	8	9	10
1. Acceptability of Corruption	–	.06*	–.11**	–.07*	.15**	–.23**	–.18**	–.09**	–.01	–.08**
2. Micro worries		–	.73***	.07***	.26***	.19***	.28***	–.08***	–.12***	–.09***
3. Macro worries			–	.18*	.17***	.43***	.41***	.02	.13***	–.10***
4. Openness to Change				–	.47***	.56***	.33***	–.06*	.03	.14***
5. Self-Enhancement					–	.19***	.26***	–.19***	.04	.13*
6. Self-Transcendence						–	.70***	.06*	–.05	–.03
7. Conservation							–	.17***	.03	–.05
8. Age								–	.16*	.04
9. Education									–	.27*
10. Income										–

Note. Level of significance: * p < .05, ** p < .01

*Table 2* presents the results of hierarchical regression analysis of the relationship between worries and the acceptability of corruption, considering age, education, and income. The coefficient of determination (6%) indicates the weak relationship of the variables. Micro worries positively relate to the acceptability of corruption, while macro worries negatively relate to it. Age negatively and income positively relate to the acceptability of corruption.

**Table 2:** The Relationship Between Worries and the Acceptability of Corruption

	The Acceptability of Corruption							
Variables	Model 1				Model 2			
	b	95% CI	SE	t	B	95% CI	SE	t
Age	–.08	[–.14; –.04]	.01	–3.25***	–.06	[–.11; –.01]	.01	–2.12*
Education	–.02	[–.07; .04]	.01	–.55	–.03	[–.08; .03]	.01	–.92
Income	.08	[.03; .14]	.01	2.91**	.08	[.03; .13]	.01	2.91**
Micro worries					.30	[.22; .37]	.04	7.69***
Macro worries					–.32	[–.40; –.24]	.04	–8.26***
F(3, 1376)	6.53***							
R^2^	.01							
F(5,1374)	18.92***							
R^2^	.06							

Note. Level of significance: * p < .05, ** p < .01, *** p < .001

*Table 3* presents the results of assessing the moderation effect of Openness to Change on the relationship between micro worries and the acceptability of corruption. The direct effects of micro worries and Openness to Change on the acceptability of corruption are statistically significant. Micro worries positively relate to the acceptability of corruption, while values of Openness to Change negatively relate to it. The effect of the interaction of micro worries and the value of Openness to Change is significant and positive for the acceptability of corruption only in the middle level of Openness to Change.

**Table 3:** The Relationship Between Micro Worries and the Acceptability of Corruption Moderated by Openness to Change Values

	The Acceptability of Corruption				
	B	95% CI	SE	Z
Micro worries	.08	[.02; .13]	.03	2.57**
Openness to Change	–.10	[–.17; –.02]	.04	–2.60**
Micro worries*Openness to Change:				
1. Low moderator score	.07	[–.01; .17]	.05	1.82
2. Middle moderator score	.08	[–.02; .13]	.03	2.57*
3. High moderator score	.07	[–.01; .14]	.04	1.81
F (3, 1376)	4.14**			
R^2^	.01			

Note. Level of significance: * p < .05, ** p < .01, *** p < .001

*Table 4* shows the results of assessing the moderation effect of Openness to Change on the relationship between macro worries and the acceptability of corruption. The direct impact of macro worries on the acceptability of corruption is statistically significant and negative. At the same time, this relationship becomes more robust with the growth of the severity of the Openness to Change, starting from the middle level.

**Table 4:** The Relationship Between Macro Worries and the Acceptability of Corruption Moderated by Openness to Change Values

	Acceptability of Corruption				
	b	95% CI	SE	z
Macro worries	–.10	[–.15; –.04]	.03	–3.55***
Openness to Change	–.07	[–.14; .01]	.04	–1.76
Macro worries*Openness to Change:				
1. Low moderator score	–.08	[–.16; .00]	.04	–1.91
2. Middle moderator score	–.10	[–.15; –.04]	.03	–3.55***
3. High moderator score	–.12	[–.19; –.05]	.04	–3.24***
F (3, 1376)		6.50***		
R^2^		.01		

Note. Level of significance: * p < .05; ** p < .01, *** p < .001

*Table 5* presents the results of assessing the moderation effect of the Self-Enhancement values in the relationship between micro worries and the acceptability of corruption. Only Self-Enhancement values significantly and positively related to the acceptability of corruption. There was no effect of the interaction of micro worries and Self-Enhancement values.

**Table 5:** The Relationship Between Micro Worries and the Acceptability of Corruption Moderated by Self-Enhancement Values

	Acceptability of Corruption				
	b	95% CI	SE	z
Micro worries	.03	[–.03; .08]	.03	.91
Self–Enhancement	.16	[.10; 22]	.03	5.45***
Micro worries*Self–Enhancement:				
1. Low moderator score	.01	[–.07; .09]	.04	.25
2. Middle moderator score	.03	[–.03; .08]	.03	.91
3. High moderator score	.04	[.04; .12]	.04	1.14
F (3, 1376)	11.11***			
R^2^	.02			

Note. Level of significance: * p < .05; ** p < .01, *** p < .001

*Table 6* presents the results of assessing the moderation effect of Self-Enhancement values in the relationship between macro worries and the acceptability of corruption. The direct effects of macro worries and Self-Enhancement values are statistically significant. Macro worries negatively relate to the acceptability of corruption, while values of Self-Enhancement positively relate to it. The effect of the interaction of macro worries and values of Self-Enhancement is statistically significant and positive for the acceptability of corruption and weakens with the increase of Self-Enhancement.

**Table 6:** The Relationship Between Macro Worries and the Acceptability of Corruption Moderated by Self-Enhancement Values

	Acceptability of Corruption				
	b	95% CI	SE	z
Macro worries	–.14	[–.19; –.09]	.03	–5.18***
Self–Enhancement	.20	[.14; .25]	.03	6.59***
Macro worries*Self–Enhancement:				
1. Low moderator score	–.152	[–.22; –.07]	.04	–3.93***
2. Middle moderator score	–.144	[–.19; –.09]	.03	–5.17***
3. High moderator score	–.141	[–.21; –.07]	.04	–3.77***
F (3, 1376)	19.54***			
R^2^	.04			

Note. Level of significance: * p < .05, ** p < .01, *** p < .001

*Table 7* presents the results of assessing the moderation effect of Self-Transcendence values on the relationship between micro worries and the acceptability of corruption. The direct effects of micro worries and Self-Transcendence values are statistically significant. Micro worries positively relate to the acceptability of corruption, while Self-Transcendence values negatively relate to it. The effect of the interaction of micro worries and Self-Transcendence values is significant and positive for the acceptability of corruption. It increases with an increase in the strength of Self-Transcendence values.

**Table 7:** The Relationship Between Micro Worries and the Acceptability of Corruption Moderated by Self-Transcendence Values

	Acceptability of Corruption			
	b	95% CI	SE	z
Micro worries	.12	[.06; .17]	.03	4.18***
Self–Transcendence	–.34	[–.40; –.27]	.04	–9.62***
Micro worries*Self–Transcendence:				
1. Low moderator score	.11	[.03; .19]	.04	2.67**
2. Middle moderator score	.12	[.06; .17]	.03	4.18***
3. High moderator score	.13	[.05; .20]	.04	3.37***
F (3, 1376)	31.64***			
R^2^	.07			

Note. Level of significance: * p < .05, ** p < .01, *** p < .001

*Table 8* presents the results of assessing the moderation effect of Self-Transcendence values on the relationship between macro worries and the acceptability of corruption. Self-transcendence values are significantly and negatively related to the acceptability of corruption. There was no effect of the interaction of macro worries and Self-Transcendence.

**Table 8:** The Relationship Between Macro Worries and the Acceptability of Corruption Moderated by Self-Transcendence Values

	Acceptability of Corruptio				
	b	95% CI	SE	z
Macro worries	-.01	[-.07; .04]	.03	-.45
Self-Transcendence	-.30	[-.37; -.23]	.04	-8.56***
Macro worries*Self-Transcendence:				
1. Low moderator score	-.04	[-.12; .03]	.04	-1.13
2. Middle moderator score	-.01	[-.07; .04]	.03	-.45
3. High moderator score	.02	[-.05; .09]	.04	.50
F (3, 1376)	26.12***			
R^2^	.05			

Note. Level of significance: * p < .05; ** p < .01, *** p < .001

*Table 9* presents the results of assessing the moderation effect of the value Conservation on the relationship between micro worries and the acceptability of corruption. The direct effect of micro worries on Conservation values is significant. Micro worries positively relate to the acceptability of corruption, while Conservation values

**Table 9:** The Relationship Between Micro Worries and the Acceptability of Corruption Moderated by Conservation Values

	Acceptability of Corruption			
	b	95% CI	SE	z
Micro worries	.13	[.07; .19]	.03	4.59***
Conservation	–.27	[–.34; –.21]	.03	–7.98***
Micro worries*Conservation:				
1. Low moderator score	.129	[.05; .21]	.04	3.15**
2. Middle moderator score	.130	[.07; .19]	.03	4.59***
3. High moderator score	.131	[.06; .20]	.04	3.53***
F (3, 1376)	21.5***			
R^2^	.05			

Note. Level of significance: * p < .05; ** p < .01, *** p < .001

negatively relate to it. The effect of the interaction of micro worries and Conservation values is significant and positive for the acceptability of corruption, which slightly increases with the growth of the severity of Conservation values.

*Table 10* shows the results of assessing the moderation effect of the value Conservation on the relationship between macro worries and the acceptability of corruption. Conservation values are significantly and negatively related to the acceptability of corruption. The effect of the interaction of macro worries and Conservation values is significant and negative for the acceptability of corruption only at the low level of Conservation values.

**Table 10:** The Relationship Between Macro Worries and the Acceptability of Corruption Moderated by Conservation Values

	Acceptability of Corruption			
	b	95% CI	SE	z
Macro worries	–.05	[–.10; .01]	.03	–1.73
Conservation	–.20	[–.27; –.13]	.03	–5.86***
Macro worries*Conservation:				
1. Low moderator score	–.10	[–.17; –.03]	.04	–2.76**
2. Middle moderator score	–.05	[–.10; .01]	.03	–1.73
3. High moderator score	.01	[–.06; .08]	.04	.20
F (3, 1376)	17.23***			
R^2^	.04			

Note. Level of significance: * p < .05; ** p < .01, *** p < .001

## Discussion

This research confirmed a suggestion about the nature of the relationship between micro worries and the acceptability of corruption. In particular, the research results show that the stronger the respondents worry about personal problems and problems of loved ones (micro worries), the stronger the acceptability of corruption. These results correspond to the idea of worry as a consequence of human suffering (Dugas et al., 1997; Lefebvre et al., 2017; Palmer et al., 2021), and that suffering people have a greater readiness for dishonest behavior (Poon et al., 2013). This is because people tend to view the suffering they experience as something they do not deserve and dishonest behavior as compensation for such injustice (Kouchaki & Desai, 2015; Mc-Parland & Eccleston, 2013; Sullivan et al., 2012). Similar results were obtained in a study by A. Mironova and A. Tatarko (Mironova & Tatarko, 2021), based on the data from the World Values Survey. It showed in examples from 15 countries that micro worries are positively related to the acceptability of corruption.

The present research also confirmed the hypothesis of the harmful nature of macro worries and the acceptability of corruption. The more the respondents worry about the situation in the country, the less they accept corruption. This result is natural, as corruption is a problem on a societal level. If people worry about the state of a country, they observe corruption as a source of the problem and reject it. Similar results were obtained by Mironova and Tatarko (2021), where it was found that worries about problems in a country (macro worries) were negatively related to the degree of acceptability of corruption.

Individual values are another critical factor in the acceptability of corruption (Mansilla, 2003; Welzel et al., 2003). That is why, in the research model, the higher-order values (Schwartz et al., 2012) were accounted as moderators. The direct connection of values with the acceptability of corruption was entirely consistent with the results of the multigroup model constructed in a similar cross-cultural study (Tatar-ko et al., 2020). To put it briefly, Self-Enhancement values positively relate to the acceptability of corruption, while values of Self-Transcendence, Openness to Change, and Conservation negatively relate to it. However, the interactive effect of worries and higher-order values on the acceptability of corruption evokes greater interest. That is why eight models were constructed for this aim, with six out of eight models demonstrating statistically significant results. These are:

(a) Openness to Change values strengthen a negative relationship between macro worries and support the negative relationship between micro worries and the acceptability of corruption only at the middle level of the severity of these values.
(b) Conservation values increase a positive relationship between micro worries and the acceptability of corruption despite the negative direct relationship with the acceptability of corruption. Macro worries negatively relate to the acceptability of corruption only at the low level of the severity of these values.
(c) Self-Enhancement values weaken a negative relationship between macro worries and the acceptability of corruption. In contrast, there was no significant effect of values on the relationship between micro worries and the acceptability of corruption.
d) Self-Transcendence values strengthen a positive relationship between micro worries and the acceptability of corruption despite a negative direct relationship with the acceptability of corruption. These values do not significantly affect the relationship between macro worries and the acceptability of corruption.

Thus, values directly relate to the acceptability of corruption and change the relationship between worries and the acceptability of corruption. The effect of values on this relationship is moderately expressed but statistically significant. In the context of worries, the inhibitory effect on the acceptability of corruption exists in the case of Openness to Change only, which strengthen a negative relationship between macro worries and the acceptability of corruption. Other values play a role as the catalyst in this relationship despite a significant direct effect on the acceptability of corruption. It has wildly unexpected results for Conservation and Self-Transcendence, which have adverse direct effects on the acceptability of corruption. Nevertheless, values can also increase a positive relationship between micro worries and the acceptability of corruption. In Schwartz’s theory, these values have a societal focus (Schwartz et al., 2012). The possible explanation for this result is that socially oriented people have high justice sensitivity (Baumert & Schmitt, 2016). However, if these people feel worried or suffer, they perceive these feelings as injustice and intend to restore them by the mechanism described in the theoretical part of this article (McParland & Eccleston, 2013; Sullivan et al., 2012).

These values and micro worries are relatively strongly expressed among Russians. Micro worries have a high value due to the country’s complex history and unstable political situation. Conservation and Self-Transcendence values dominate values of Openness to Change and Self-Enhancement among Russians (Lebedeva & Tatarko, 2018). In combination with other phenomena, this may explain Russia’s traditionally high level of corruption.

## Conclusion

The main theoretical contribution of this research is that it explains how some negative social phenomena in society, particularly corruption, can be explained using the concept of the relationship between suffering and dishonest behavior. The research results demonstrate that people who are very worried about themselves and their loved ones are more likely to accept corruption in everyday life. At the same time, worrying about society induces people to vindicate corruption less. Besides worries evoked by suffering, the acceptability of corruption is related to values, particularly Schwartz`s higher-order values. Higher-order values not only directly relate to the acceptability of corruption, but also can change the relationship between worries and the acceptability of corruption. It can be concluded that increasing micro worries (about one’s job, loved ones, health, future, etc.) evoked by difficult life circumstances, against the backdrop of clearly expressed the higher-order values Conservation and Self-Transcendence, can lead to high risks of everyday corruption. We see the opposite situation when citizens worry about global problems (macro worries) (Inglehart & Welzel, 2005) against the backdrop of clearly expressed higher-order values Openness to Change, leading to decreased corruption risks. Improving the standard of living and reducing the number of political disturbances, conflicts, and wars, a clear vision of the future, will abate people’s worries and the readiness to accept everyday corruption. The results also have practical applications, for instance, in selecting civil servants. Analyzing a combination of micro and macro worries with higher-order values allows us to deduce whether a candidate for public office or a public servant is predisposed to corrupt behavior and risks being involved in dishonest behavior.

## Limitations and Future Perspectives

Even though macro/micro worries and higher-order values as predictors of the acceptability of corruption are well justified, this research has a correlation research design. An experiment would have to be conducted to prove a causal relationship. However, experimenting is difficult due to the inclusion of values in the study, the manipulation of which seems impossible. It is also essential to understand that the study was conducted in Russia, which has relatively high levels of corruption, a complex history, and a unique combination of core values. In this regard, it is important to recheck these results in other countries with lower levels of corruption and a different combination of basic values. Further cross-cultural research might be conducted in this direction.

Future research could also focus on developing and testing intervention-based approaches, such as educational or policy programs aimed at reducing the acceptability of corruption by addressing individual worries and promoting value-based decision-making. Such interventions may help foster more resilient anti-corruption attitudes at both individual and societal levels.

Moreover, exploring additional psychological factors that may moderate the relationship between worries and the acceptability of corruption would be valuable. Investigating the roles of institutional trust, personal morality, and perceived fairness could deepen understanding of the complex mechanisms underlying attitudes toward corruption.

## References

[ref1] Bastian, B., Jetten, J., & Fasoli, F. (2011). Cleansing the soul by hurting the flesh: The guilt-reducing effect of pain. Psychological Science, 22(3), 334–335. 10.1177/0956797610397058 21245493

[ref2] Bastian, B., Jetten, J., Hornsey, M.J., & Leknes, S. (2014). The positive consequences of pain: A bio-psychosocial approach. Personality and Social Psychology Review, 18(3), 256–279. 10.1177/1088868314527831 24727972

[ref3] Baumert, A., Schmitt, M. (2016). Justice sensitivity. In C. Sabbagh, & M. Schmitt (Eds.), Handbook of social justice theory and research (pp. 161–180). Springer. 10.1007/978-1-4939-3216-0_9

[ref4] Boehnke, K., Schwartz, Sh., Stromberg, C., & Sagiv, L. (1998). The structure and dynamics of worry: Theory, measurement, and cross-national replications. Journal of Personality, 66(5), 745–782. 10.1111/1467-6494.00031 9802232

[ref5] Bosco, B. (2016). Old and new factors affecting corruption in Europe: Evidence from panel data. Economic Analysis and Policy, 51, 66–85. 10.1016/j.eap.2016.06.002

[ref6] Chattopadhyay, S. (2013). Corruption in healthcare and medicine: Why should physicians and bio-ethicists care and what should they do. Indian Journal of Medical Ethics, 10(3), 153–159. 10.20529/IJME.2013.049 23912727

[ref7] Davis, J.H., & Ruhe, J.A. (2003). Perceptions of country corruption: Antecedents and outcomes. Journal of Business Ethics, 43, 275–288. 10.1023/A:1023038901080

[ref8] Dugas, M.J., Freeston, M.H., & Ladouceur, R. (1997). Intolerance of uncertainty and problem orientation in worry. Cognitive Therapy and Research, 21, 593–606. 10.1023/A:1021890322153

[ref9] Dugas, M.J., Letarte, H., Rhéaume, J., Freestone, M.H., & Ladouceur, R. (1995). Worry and problem solving: Evidence of a specific relationship. Cognitive Therapy and Research, 19, 109–120. 10.1007/BF02229679

[ref10] Eysenck, M.W. (1984). Anxiety and the worry process. Bulletin of the Psychonomic Society, 22, 545–548. 10.3758/BF03333903

[ref11] Filipkowski, K.B., Smyth, J.M., Rutchick, A.M., Santuzzi, A.M., Adya, M., Petrie, K.J., & Kaptein, A.A. (2010). Do healthy people worry? Modern health worries, subjective health complaints, perceived health, and health care utilization. International Journal of Behavioral Medicine, 17, 182–188. 10.1007/s12529-009-9058-0 19763842

[ref12] Furnham, A. (2003). Belief in a just world: Research progress over the past decade. Personality and Individual Differences, 34(5), 795–817. 10.1016/S0191-8869(02)00072-7

[ref13] Graeff, P., & Mehlkop, G. (2003). The impact of economic freedom on corruption: Different patterns for rich and poor countries. European Journal of Political Economy, 19(3), 605–620. 10.1016/S0176-2680(03)00015-6

[ref14] Griffin, M., & Prior, M. (1990). Young people and the nuclear threat. Journal of Youth Studies, 7(2), 40–43.

[ref15] Gupta, S., Davoodi, H., & Alonso-Terme, R. (2002). Does corruption affect income inequality and poverty? Economics of Governance, 3(1), 23–45. 10.1007/s101010100039

[ref16] Hamilton, S.B., Lynch, R.S., Naginey, J.L., Peters, K.A., & Piske, K.R. (1989). Relationships between the life values of US College students and their cognitive/affective responses to the threat of nuclear war. Journal of Adolescence, 12(1), 55–68. 10.1016/0140-1971(89)90089-4 2708601

[ref17] Heyneman, S.P. (2004). Education and corruption. International Journal of Educational Development, 24(6), 637–648. 10.1016/j.ijedudev.2004.02.005

[ref18] Hickman, S.E., Tilden, V.P., & Tolle, S.W. (2004). Family perceptions of worry, symptoms, and suffering in the dying. Journal of Palliative Care, 20(1), 20–27. 10.1177/082585970402000105 15132072

[ref19] Husted, B. (1999). Wealth, culture and corruption. Journal of International Business Studies, 30(2), 339–346. 10.1057/palgrave.jibs.8490073

[ref20] Inbar, Y., Pizarro, D.A., Gilovich, T., & Ariely, D. (2013). Moral masochism: On the connection between guilt and self-punishment. Emotion, 13(1), 14–18. 10.1037/a0029749 22985340

[ref21] Inglehart, R., & Welzel, C. (2005). Modernization, cultural change, and democracy: The human development sequence. Cambridge University Press. 10.1017/CBO9780511790881

[ref22] Jong-Sung, Y., & Khagram, S. (2005). A comparative study of inequality and corruption. American Sociological Review, 70(1), 136–157. 10.1177/000312240507000309

[ref23] Kouchaki, M., & Desai, S.D. (2015). Anxious, threatened, and also unethical: How anxiety makes individuals feel threatened and commit unethical acts. Journal of Applied Psychology, 100(2), 360–375. 10.1037/a0037796 25243997

[ref24] Kravtsova, M., Oshchepkov, A., & Welzel, C. (2017). Values and corruption: Do postmaterialists justify bribery? Journal of Cross-Cultural Psychology, 48(2), 225–242. 10.1177/0022022116677579

[ref25] Kreikebaum, H. (2008). Corruption as a moral issue. Social Responsibility Journal, 4 (1–2), 82–88. 10.1108/17471110810856857

[ref26] Kubiak, A. (2001). Corruption in everyday experience: Report on a survey. Warsaw Institute of Public Affairs.

[ref27] Lambsdorff, J.G. (2006). Causes and consequences of corruption: What do we know from a cross-section of countries? In S. Rose-Ackerman (Ed.), International handbook on the economics of corruption (pp. 3–51). Edward Elgar. 10.4337/9781847203106.00007

[ref28] Lebedeva, N.M., & Tatarko, A.N. (2018). Basic values in Russia: Their dynamics, ethnocultural differences, and relation to economic attitudes. Psychology in Russia: State of the Art, 11(3), 36–52. 10.11621/pir.2018.0303

[ref29] Lee, W.S., & Guven, C. (2013). Engaging in corruption: The influence of cultural values and contagion effects at the microlevel. Journal of Economic Psychology, 39, 287–300. 10.1016/j.joep.2013.09.006

[ref30] Lefebvre, J.C., Jensen, M.P., & Trant, D.A. (2017). The effects of manipulating worry and happiness on the experience of acute pain and worry about pain. Cognitive Therapy and Research, 41, 787–798. 10.1007/s10608-017-9854-9

[ref31] Mansilla, H.C.F. (2003). La mentalidad tradicional como obstáculo a la democratización en el caso bo-liviano [The traditional mentality as an obstacle to democratization in Bolivia]. Psicologia Política, 26, 25–40.

[ref32] McParland, J.L., & Eccleston, C. (2013). “It’s not fair”: Social justice appraisals in the context of chronic pain. Current Directions in Psychological Science, 22(6), 484–489. 10.1177/0963721413496811

[ref33] Milgram, S. (1963). Behavioral study of obedience. The Journal of Abnormal and Social Psychology, 67(4), 371–378. 10.1037/h0040525 14049516

[ref34] Mironova, A.A., & Tatarko, A.N. (2021). Psychological causes of corruption: The role of worries. Journal of Economic Sociology, 22(1), 11–34. 10.17323/1726-3247-2021-1-11-34

[ref35] Molina, S., & Borkovec, T.D. (1994). The Penn State Worry Questionnaire: Psychometric properties and associated characteristics. In G.C. Davey & F. Tallis (Eds), Worry: Perspectives on Theory, Assessment, and Treatment (pp. 265–283). Wiley.

[ref36] Nelissen, R., & Zeelenberg, M. (2009). When guilt evokes self-punishment: Evidence for the existence of a Dobby effect. Emotion, 9(1), 118–122. 10.1037/a0014540 19186924

[ref37] Palmer, C.A., Oosterhoff, B., & Gentzler, A.L. (2021). Don’t worry, be happy: Associations between worry and positive emotion regulation. International Journal of Cognitive Therapy, 14, 417–435. https://doi.org/10.1007/s41811-020-00081-8

[ref38] Park, H. (2003). Determinants of corruption: A cross-national analysis. The Multinational Business Review, 11(2), 29–48. 10.1108/1525383X200300010

[ref39] Poon, K.-T., Chen, Z., & De Wall, C.N. (2013). Feeling entitled to more: Ostracism increases dishonest behavior. Personality and Social Psychology Bulletin, 39(9), 1227–1239. 10.1177/0146167213493187 23812925

[ref40] Schwartz, S.H., Butenko, T.P., Sedova, D.S., & Lipatova, A.S. (2012). Utochnennaia teoriia bazovykh individual’nykh tsennostei: primenenie v rossii [A refined theory of basic personal values: Application in Russia]. Psikhologiia. Zhurnal Vysshei shkoly ekonomiki [Psychology. Journal of the Higher School of Economics], 9(1), 43–70. https://psy-journal.hse.ru/data/2013/10/30/1283379255/Schwartz_et_al_9-02pp43-70.pdf

[ref41] Schwartz, S.H. (1992). Universals in the content and structure of values: Theoretical advances and empirical tests in 20 countries. Advances in Experimental Social Psychology, 25, 1–65. 10.1016/S0065-2601(08)60281-6

[ref42] Schwartz, S.H., Cieciuch, J., Vecchione, M., Davidov, E., Fischer, R., Beierlein, C., Ramos, A., Verkasalo, M., Lönnqvist, J.-E., Demirutku, K., Dirilen-Gumus, O., & Konty, M. (2012). Refining the theory of basic individual values. Journal of Personality and Social Psychology, 103, 663–688. 10.1037/a0029393 22823292

[ref43] Siddique, H.I., LaSalle-Ricci, V.H., Glass, C.R., Arnkoff, D.B., & Diaz, R.J. (2006). Worry, optimism, and expectations as predictors of anxiety and performance in the first year of law school. Cognitive Therapy and Research, 30, 667–676. 10.1007/s10608-006-9080-3

[ref44] Sullivan, M.J.L., Scott, W., & Trost, Z. (2012). Perceived injustice: A risk factor for problematic pain outcomes. Clinical Journal of Pain, 28(6), 484–488. 10.1097/ajp.0b013e3182527d13 22673480

[ref45] Sumah, S. (2017). Corruption: Causes and consequences. In V. Bobek (Ed.), Trade and global market. InTech. 10.5772/intechopen.70966

[ref46] Svensson J. (2005). Eight questions about corruption. The Journal of Economic Perspectives, 19(3), 19–42. 10.1257/089533005774357860

[ref47] Tallis, F., Davey, G.C.L., & Bond, A. (1994). The Worry Domains Questionnaire. In: G.C.L Davey & Tallis (Eds.), Worry: perspectives on theory, assessment, and treatment (pp. 285-297). Wiley.

[ref48] Tanner, C., Linder, S., & Sohn, M. (2022). Does moral commitment predict resistance to corruption? Experimental evidence from a bribery game. PLoS One, 17(1), 1–22. 10.1371/journal.pone.0262201 PMC8752004 35015764

[ref49] Tatarko, A.N., & Mironova, A.A. (2016). Values and attitudes towards corruption: A cross-cultural study in four European countries. SSRN Electronic Journal, 1–22. 10.2139/ssrn.2791414

[ref50] Tatarko, A.N., Maklasova, E.V., & Van de Vliert, E. (2022). Climato-economic context of regional crime and corruption across the Russian Federation. Environment and Behavior, 54(3), 575–596. 10.1177/00139165211060522

[ref51] Tatarko, A.N., & Mironova, A.A. (2015). Values and trust as factors of attitude toward corruption. Psycho logy in Economics and Management, 7(2), 96–110. 10.17150/2225-7845.2015.7(2).96-110

[ref52] Tatarko, A.N., Mironova, A.A., Gari, A., & van de Vijver, F. (2020). The relationship between human values and acceptability of corruption in Russia and Greece. Psychology in Russia. State of the Art, 13(3), 79–95. 10.11621/pir.2020.0306

[ref53] Torgler, B., & Valev, N.T. (2006). Corruption and age. Journal of Bioeconomics, 8(2), 133–145. 10.1007/s10818-006-9003-0

[ref54] Torgler, B., Valev, N.T. (2010). Gender and public attitude toward corruption and tax evasion. Contemporary Economic Policy, 28(4), 554–568. 10.1111/j.1465-7287.2009.00188.x

[ref55] Welzel, C., Inglehart, R., & Klingemann, H. (2003). The theory of human development: A cross- cultural analysis. European Journal of Political Research, 42, 341–379. 10.1111/1475-6765.00086

[ref56] Zimbardo, P.G. (1969). The human choice: Individuation, reason, and order versus deindividuation, impulse, and chaos. In W.J. Arnold & D. Levine (Eds.), Nebraska Symposium on Motivation (pp. 237–307). University of Nebraska Press.

[ref57] Tereshonok, T.A. (2016). Korruptsiia v sfere zdravookhraneniia [Corruption in the healthcare sector]. Plekhanovskii barometr [The Plekhanov Barometer Journal], 4, 21–24.

